# The Persimmon

**Published:** 1867-11

**Authors:** 


*The Persimmon.*—This old friend of our boyhood, that served our infant mouths so many shabby tricks, by pretending to be ripe when it was not, and then set us to premature and painful whistling, has lately come out as a first class astringent. Every boy, we are sure, will endorse it for that. Dr. Mettauer, a well known physician and surgeon of Prince Edward County Virginia, has been discoursing on its value in the *Boston Medical and Surgical Journal.* The unripe fruit is used in the form of tincture, syrup, or infusion. Good results have been obtained from it in Cholera infantum, diarrhea, dysentery, menorrhagia, gleet, bronchorrhœa and coryza. The Doctor says that mixed with rhubarb, it is a good vermifuge.

